# Detection and phylogenetic classification of *Neoehrlichia mikurensis* in rodents from the region of Liupan Mountain, China

**DOI:** 10.3389/fmicb.2024.1409593

**Published:** 2024-07-04

**Authors:** Xiaodong Wang, Bo Pang, Zengqiang Kou, Jiaqi Zhao, Yi Yan, Tan Chen, Liping Yang

**Affiliations:** ^1^Department of Epidemiology, School of Public Health, Cheeloo College of Medicine, Shandong University, Jinan, China; ^2^Shandong Center for Disease Control and Prevention, Jinan, China; ^3^Jingyuan Center for Disease Control and Prevention, Guyuan, China

**Keywords:** *Neoehrlichia mikurensis*, rodents, detection, phylogenetic analysis, Liupan Mountain

## Abstract

*Neoehrlichia mikurensis* (*N. mikurensis*) is an emerging tick-borne pathogen that can cause neoehrlichiosis. Rodents are considered the major host for *N. mikurensis*. Currently, *N. mikurensis* has been detected in rodents in several studies from China and other countries. However, no research on *N. mikurensis* infection in rodents has been reported in the Liupan mountain region. The region of Liupan Mountain, located in northwestern China, is the center of the triangle formed by the cities of Xi’an, Yinchuan, and Lanzhou, with multiple tourist sites in the region. To survey whether there is *N. mikurensis* in hosts, rodents were captured in this region in September 2020. A nested polymerase chain reaction was used to detect the DNA of *N. mikurensis*, followed by nucleotide sequencing and phylogenetic analysis. In the region, among 88 rodents, 3 rodents were detected positive for *N. mikurensis*, a detection rate of 3.4%. Based on phylogenetic analysis of the partial *groEL* gene sequences, *N. mikurensis* from rodents in Liupan Mountain clustered in the same evolutionary branch with those found in rodents from Japan, Russia, and northeastern China, and also in ticks and clinical cases from Heilongjiang Province in northeastern China.

## 1 Introduction

*Neoehrlichia mikurensis* (*N. mikurensis*), an emerging pathogen belonging to the family *Anaplasmataceae* (Kawahara et al., 2004), was first detected in *Ixodes ricinus* in the Netherlands in 1999 (Schouls et al., 1999). Currently, *N. mikurensis* has been reported among a variety of animals, such as rodents (Obiegala et al., 2014), hedgehogs (Földvári et al., 2014), and a dog (Diniz et al., 2011). Of these, rodents can be considered to be the major host for *N. mikurensis.*

*N. mikurensis* in rodents has been reported in several countries, such as South Korea (Jha et al., 2018), Germany (Galfsky et al., 2019), Sweden (Andersson and Råberg, 2011), Japan (Kenji et al., 2007), Hungary (Szekeres et al., 2015), and Russia (Rar et al., 2010). In 2003, Pan et al. (2003) found the pathogen in *Rattus norvegicus* from Guangzhou, China. Subsequently, it was also found in rodents from other provinces in China, such as Yunnan (Du et al., 2018), Jilin (Li et al., 2013) and Liaoning provinces (Wang et al., 2019). Since the first human case of neoehrlichiosis was reported in 2010 (Welinder-Olsson et al., 2010), more and more human infections with *N. mikurensis* have been reported globally (Pekova et al., 2011; Li et al., 2012; Welc-Faleciak et al., 2014; Quarsten et al., 2017; Boyer et al., 2021; Hoper et al., 2021; Lenart et al., 2021; Markowicz et al., 2021; Gonzalez-Carmona et al., 2023; Gynthersen et al., 2023).

However, no research on neoehrlichiosis or *N. mikurensis* infection in hosts has been reported in the region of Liupan Mountain, China. So, detecting whether there is *N. mikurensis* in rodents is of great significance for assessing the risk of *N. mikurensis* infection in residents. Silaghi et al. (2012) detected *N. mikurensis* in the liver, spleen, kidney, blood, transudate, and skin tissues of rodents, with the highest detection rate in the kidney. Therefore, in this study, we used nested PCR to detect the presence of *N. mikurensis* in rodent kidney organs in the region.

## 2 Materials and methods

### 2.1 Study area and sample collection

The Liupan Mountain Nature Reserve (106°09′-106°30′E, 35°15′-35°41′N), located in northwestern China, is the center of the triangle formed by the cities of Xi’an, Yinchuan, and Lanzhou, with multiple tourist sites in the region.

In September 2020, rodents were captured by night trapping method at Laolongtan Scenic Spot, Huanghua Town, and Xingsheng Town in the region. After the captured rodents were brought back to the laboratory, they were species-identified based on their morphological characteristics (Lu, 1982), and then dissection was performed to obtain the kidney tissues, which were stored in 2 mL cryotubes at −80°C.

### 2.2 DNA extraction and detection of *Neoehrlichia mikurensis*

Approximately 25 mg of kidney tissue was taken under aseptic operation. DNA was extracted according to the instructions of a Tissue DNA Isolation Kit (Magnetic Beads) (Jiangsu Bioperfectus Technologies Co., Ltd., Jiangsu, China) and stored at −40°C.

The *groEL* gene of *N. mikurensis* was amplified by nested PCR. The primers were used as reported by Li et al. (2013) and synthesized by Shanghai BioGerm Medical Technology Co., Ltd. (Shanghai, China). The final amplification product target fragment size was 891 bp. For the first round of the PCR amplification, the reaction mix was prepared as follows: 12.5 μL of Premix Taq^™^ (TaKaRa Taq^™^ Version 2.0), 0.5 μL each of CNM-out 1 and CNM-out 2 (10 μmol/L), DNA template 3 μL, ultrapure water 8.5 μL. The reaction conditions were: 94°C pre-denaturation 5 min; 94°C denaturation 30 s, 53°C annealing 30 s, 72°C extension 1 min 30 s, for a total of 40 cycles; 72°C final extension 5 min. For the second round of PCR amplification, the reaction mix was prepared as follows: Premix Taq^™^ (TaKaRa Taq^™^ Version 2.0) 12.5 μL, 1 μL each of CNM-in 1 and CNM-in 2 (10 μmol/L), 0.5 μL of first-round product, and 10 μL of ultrapure water. The reaction conditions were the same as those of the first-round reaction, except for the final extension at 72°C for 1 min. The PCR products were separated using electrophoresis on 1.5% agarose gels, with GelRed^™^ Nucleic Acid Gel Stain (Biotium, America) and visualized under Gel Doc^™^ XR+ Imaging System (Bio-Rad, United States).

### 2.3 Homology and phylogenetic analyses

The positive products were sequenced bidirectionally by Shanghai BioGerm Medical Technology Co., Ltd. (Shanghai, China). Sequence assembly and manual correction were performed using SeqMan software (Version 7.1, Lasergene, DNASTAR, Madison, WI, United States). One of the assembled sequences was uploaded to GenBank database, and used for homology and phylogenetic analyses. After performing blast alignment of the selected sequence at the National Center for Biotechnology Information, sequences with high homology were downloaded. In addition, sequences from selected geographical origins and hosts were also downloaded in this study to better analyze the evolutionary information of the *groEL* of *N. mikurensis*. The *groEL* sequences of *Anaplasma phagocytophilum* (AF033101) and *Ehrlichia chaffeensis* (L10917) were used as outgroups. Based on MEGA X (Kumar et al., 2018), model with the lowest BIC scores (Bayesian Information Criterion) was considered as the best-fit substitution model. The phylogenetic tree was constructed by the maximum likelihood method based on the best-fit substitution model (Tamura 3-parameter+G + I) (Tamura, 1992) using MEGA X (Kumar et al., 2018) and tested with 1,000 bootstrap replications. Sequences were aligned with MegAlign software (Version 7.1, Lasergene, DNASTAR, Madison, WI, United States), and the percent identities were calculated with the same software.

## 3 Results

### 3.1 Sampling and PCR detection

Among 88 rodents captured in the region, 42 were form Laolongtan Scenic Spot, 27 from Huanghua Town, and 19 from Xingsheng Town. *Apodemus peninsulae* (36.4%, 32/88), *Cricetus migratorius* (26.1%, 23/88), *Tscherskia triton* (21.6%, 19/88), *Apodemus agrarius* (11.4%, 10/88), *Niviventer confucianus* (2.3%, 2/88), *Eozapus setchuanus* (1.1%, 1/88), and *Mus musculus* (1.1%, 1/88) were identified.

Three samples were positive for *N. mikurensis* positive, including two *A. peninsulae* (LPM-R14 and LPM-R25) and one *T. triton* (LPM-R37).

### 3.2 Homology and phylogenetic analyses

**Figure 1:**
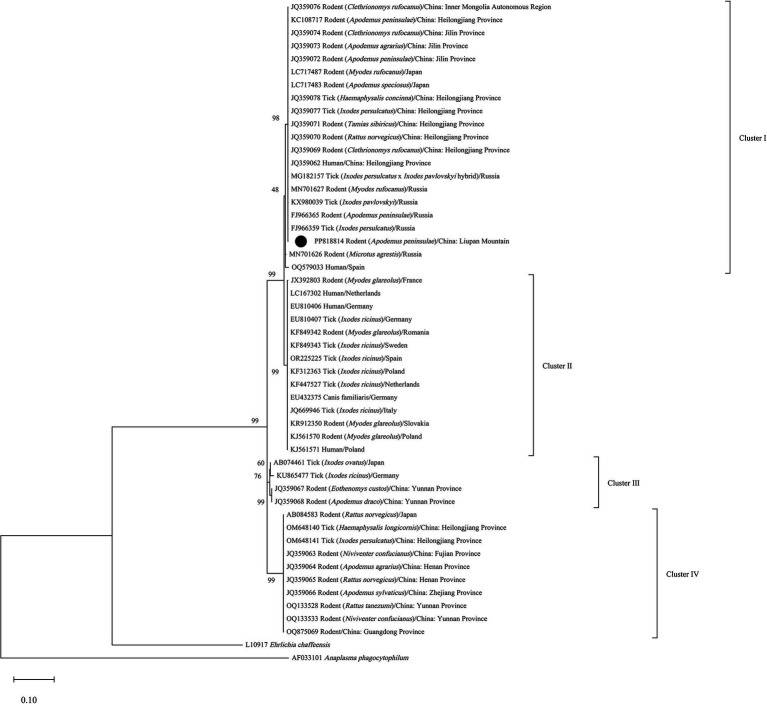
Phylogenetic tree of *N. mikurensis* inferred from partial fragments of the *groEL* gene sequences. •, Sequence obtained in the study.

The similarity of the three *N. mikurensis* sequences obtained in this study was greater than 99%. The sequence of sample LPM-R14 was uploaded to GenBank database (accession number, PP818814). Based on phylogenetic analysis of the partial *groEL* gene sequences, four evolutionary branches of *N. mikurensis* were reconstructed (Figure 1). The overall tree topology was consistent with Li et al. (2013), in particular the subdivision of *N. mikurensis* into four main clades (Clusters I-IV). The sequence (PP818814) from *A. peninsulae* clustered in Cluster I with those found in rodents from Japan (LC717483, LC717487), Russia (MN701627, FJ966365), and northwestern China, such as Inner Mongolia Autonomous Region (JQ359076), Heilongjiang (JQ359069, JQ359070, JQ359071, KC108717) and Jilin provinces (JQ359072, JQ359073, JQ359074). Moreover, Cluster I also included *N. mikurensis* infection in ticks from Russia (MG182157, KX980039, FJ966359) and Heilongjiang Province (JQ359077, JQ359078), and clinical cases of neoehrlichiosis from Heilongjiang Province (JQ359062). The homologies of PP818814 and sequences of Clusters I-IV in the phylogenetic tree were shown in Supplementary Tables S1–S3.

## 4 Discussion

In this study, *N. mikurensis* was detected for the first time in rodents from the region of Liupan Mountain. The detection rate of *N. mikurensis* was 3.4% in the region, which was similar to Zhejiang (3.7%) and Henan provinces (3.9%), but lower than Jilin Province (13.8%), China (Li et al., 2013). Our findings indicate that sequences of *N. mikurensis* from rodents in Liupan Mountain clustered in the same evolutionary branch with those found in rodents, ticks, and human cases from Heilongjiang Province. Liupan Mountain is the center of the triangle formed by the cities of Xi’an, Yinchuan, and Lanzhou, and there are multiple tourist attractions in the region. Therefore, they indicate that there is a potential risk of *N. mikurensis* infection to the residents in Liupan Mountain. Currently, *N. mikurensis* is mainly detected by nested PCR or qPCR, and there is a lack of methods for its serologic detection (Wass et al., 2019). Due to the few reported human cases in China, limited detection methods, and insufficient understanding of the clinical manifestations of neoehrlichiosis by physicians, misdiagnosis or missed diagnosis may occur.

Based on phylogenetic analysis of the *groEL* and 16S rRNA genes, Li et al. (2013) concluded that *N. mikurensis* has four evolutionary branches (Clusters I-IV) that are related to geographic origins, with sequences from northeastern China and the Asian part of Russia clustering in Cluster I. Subsequently, Sun et al. (2023) detected sequences in ticks from northeastern China clustered in Cluster IV. The phylogenetic tree inferred in this study showed that the sequence detected in the case from Spain clustered in Cluster I. This further indicate that geographical distribution and genetic variation are not necessarily concordant, and there are at least two genetic lineages of *N. mikurensis* currently in China.

In conclusion, *N. mikurensis* was found in *A. peninsulae* and *T. triton* in Liupan Mountain, and PP818814 sourced from the region clustered into the same evolutionary branch with *N. mikurensis* infection in rodents from Japan, Russia, and northeastern China, also with the pathogen in ticks and human cases from Heilongjiang Province. They indicate that there is a potential risk of *N. mikurensis* infection to residents in the region. Further studies should be conducted to detect the presence and abundance of *N. mikurensis* in ticks from the same area. And the ability of physicians to diagnose and differential diagnose neoehrlichiosis should be improved to identify whether there is the infection of *N. mikurensis* in local residents.

## Data availability statement

The datasets presented in this study can be found in online repositories. The names of the repository/repositories and accession number(s) can be found in the article/Supplementary material.

## Ethics statement

According to *Regulation on the Management of Vector Prevention and Control* issued by National Health Commission of the People’s Republic of China and Integrated Strategy for Vector Prevention and Control included in *Healthy China 2030 Planning Framework*, rats trapped is an activity for vector control. Therefore, ethical approval was not required in the study.

## Author contributions

XW: Conceptualization, Data curation, Formal analysis, Investigation, Visualization, Writing – original draft, Writing – review & editing. BP: Resources, Writing – original draft. ZK: Writing – review & editing, Supervision. JZ: Writing – original draft, Investigation. YY: Writing – original draft, Resources. TC: Resources, Writing – original draft. LY: Writing – review & editing, Supervision.
