# Supplementary material for: Detection and phylogenetic classification of Neoehrlichia mikurensis in rodents from the region of Liupan Mountain, China
Source: Front Microbiol. 2024 Jul 4;15:1409593. doi: 10.3389/fmicb.2024.1409593 (PMC11255843; doi:10.3389/fmicb.2024.1409593)
Supplement: Supplementary file 1 [file Table_1.DOCX]

**Supplementary Table 1.** The homology between the *groEL* gene sequences of *N. mikurensis* in Cluster I in the phylogenetic tree.

|  | 1 | 2 | 3 | 4 | 5 | 6 | 7 | 8 | 9 | 10 | 11 | 12 | 13 | 14 | 15 | 16 | 17 | 18 | 19 | 20 | 21 |
| --- | --- | --- | --- | --- | --- | --- | --- | --- | --- | --- | --- | --- | --- | --- | --- | --- | --- | --- | --- | --- | --- |
| 1. JQ359076 Rodent (*C. rufocanus*)/China: Inner Mongolia Autonomous Region |  |  |  |  |  |  |  |  |  |  |  |  |  |  |  |  |  |  |  |  |  |
| 2. KC108717 Rodent (*A. peninsulae*)/China: Heilongjiang Province | 100.0 |  |  |  |  |  |  |  |  |  |  |  |  |  |  |  |  |  |  |  |  |
| 3. JQ359074 Rodent (*C. rufocanus*)/China: Jilin Province | 100.0 | 100.0 |  |  |  |  |  |  |  |  |  |  |  |  |  |  |  |  |  |  |  |
| 4. JQ359073 Rodent (*A. agrarius*)/China: Jilin Province | 100.0 | 100.0 | 100.0 |  |  |  |  |  |  |  |  |  |  |  |  |  |  |  |  |  |  |
| 5. JQ359072 Rodent (*A. peninsulae*)/China: Jilin Province | 100.0 | 100.0 | 100.0 | 100.0 |  |  |  |  |  |  |  |  |  |  |  |  |  |  |  |  |  |
| 6. LC717487 Rodent (*M. rufocanus*)/Japan | 99.9 | 99.9 | 99.9 | 99.9 | 99.9 |  |  |  |  |  |  |  |  |  |  |  |  |  |  |  |  |
| 7. LC717483 Rodent (*A. speciosus*)/Japan | 99.9 | 99.9 | 99.9 | 99.9 | 99.9 | 100.0 |  |  |  |  |  |  |  |  |  |  |  |  |  |  |  |
| 8. JQ359078 Tick (*H. concinna*)/China: Heilongjiang Province | 99.9 | 99.9 | 99.9 | 99.9 | 99.9 | 99.3 | 99.3 |  |  |  |  |  |  |  |  |  |  |  |  |  |  |
| 9. JQ359077 Tick (*I. persulcatus*)/China: Heilongjiang Province | 99.9 | 99.9 | 99.9 | 99.9 | 99.9 | 99.3 | 99.3 | 100.0 |  |  |  |  |  |  |  |  |  |  |  |  |  |
| 10. JQ359071 Rodent (*T. sibiricus*)/China: Heilongjiang Province | 99.9 | 99.9 | 99.9 | 99.9 | 99.9 | 99.3 | 99.3 | 100.0 | 100.0 |  |  |  |  |  |  |  |  |  |  |  |  |
| 11. JQ359070 Rodent (*R. norvegicus*)/China: Heilongjiang Province | 99.9 | 99.9 | 99.9 | 99.9 | 99.9 | 99.3 | 99.3 | 100.0 | 100.0 | 100.0 |  |  |  |  |  |  |  |  |  |  |  |
| 12. JQ359069 Rodent (*C. rufocanus*)/China: Heilongjiang Province | 99.9 | 99.9 | 99.9 | 99.9 | 99.9 | 99.3 | 99.3 | 100.0 | 100.0 | 100.0 | 100.0 |  |  |  |  |  |  |  |  |  |  |
| 13. JQ359062 Human/China: Heilongjiang Province | 99.9 | 99.9 | 99.9 | 99.9 | 99.9 | 99.3 | 99.3 | 100.0 | 100.0 | 100.0 | 100.0 | 100.0 |  |  |  |  |  |  |  |  |  |
| 14. MG182157 Tick (*I. persulcatus* x *I. pavlovskyi* hybrid)/Russia | 99.9 | 99.9 | 99.9 | 99.9 | 99.9 | 99.3 | 99.3 | 100.0 | 100.0 | 100.0 | 100.0 | 100.0 | 100.0 |  |  |  |  |  |  |  |  |
| 15. MN701627 Rodent (*M. rufocanus*)/Russia | 99.9 | 99.9 | 99.9 | 99.9 | 99.9 | 99.3 | 99.3 | 100.0 | 100.0 | 100.0 | 100.0 | 100.0 | 100.0 | 100.0 |  |  |  |  |  |  |  |
| 16. KX980039 Tick (*I. pavlovskyi*)/Russia | 99.9 | 99.9 | 99.9 | 99.9 | 99.9 | 99.3 | 99.3 | 100.0 | 100.0 | 100.0 | 100.0 | 100.0 | 100.0 | 100.0 | 100.0 |  |  |  |  |  |  |
| 17. FJ966365 Rodent (*A.* peninsulae)/Russia | 99.9 | 99.9 | 99.9 | 99.9 | 99.9 | 99.3 | 99.3 | 100.0 | 100.0 | 100.0 | 100.0 | 100.0 | 100.0 | 100.0 | 100.0 | 100.0 |  |  |  |  |  |
| 18. FJ966359 Tick (*I. persulcatus*)/Russia | 99.9 | 99.9 | 99.9 | 99.9 | 99.9 | 99.3 | 99.3 | 100.0 | 100.0 | 100.0 | 100.0 | 100.0 | 100.0 | 100.0 | 100.0 | 100.0 | 100.0 |  |  |  |  |
| 19. ⚫PP818814 Rodent (*A. peninsulae*)/China: Liupan Mountain | 100.0 | 100.0 | 100.0 | 100.0 | 100.0 | 100.0 | 100.0 | 100.0 | 100.0 | 100.0 | 100.0 | 100.0 | 100.0 | 100.0 | 100.0 | 100.0 | 100.0 | 100.0 |  |  |  |
| 20. MN701626 Rodent (*M. agrestis*)/Russia | 99.2 | 99.2 | 99.2 | 99.2 | 99.2 | 98.7 | 98.7 | 99.3 | 99.3 | 99.3 | 99.3 | 99.3 | 99.3 | 99.4 | 99.3 | 99.3 | 99.3 | 99.3 | 99.3 |  |  |
| 21. OQ579033 Human/Spain | 98.8 | 98.8 | 98.8 | 98.8 | 98.8 | 98.1 | 98.1 | 98.8 | 98.8 | 98.8 | 98.8 | 98.8 | 98.8 | 98.9 | 98.9 | 98.9 | 98.9 | 98.9 | 98.8 | 99.4 |  |

⚫, Sequence obtained in the study
