# Supplementary material for: Detection and phylogenetic classification of Neoehrlichia mikurensis in rodents from the region of Liupan Mountain, China
Source: Front Microbiol. 2024 Jul 4;15:1409593. doi: 10.3389/fmicb.2024.1409593 (PMC11255843; doi:10.3389/fmicb.2024.1409593)
Supplement: Supplementary file 2 [file Table_2.DOCX]

**Supplementary Table 2.** The homologies of PP818814 and sequences of Cluster Ⅱ in the phylogenetic tree.

|  | 1 | 2 | 3 | 4 | 5 | 6 | 7 | 8 | 9 | 10 | 11 | 12 | 13 | 14 | 15 |
| --- | --- | --- | --- | --- | --- | --- | --- | --- | --- | --- | --- | --- | --- | --- | --- |
| 1. ⚫PP818814 Rodent (*A. peninsulae*)/China: Liupan Mountain |  |  |  |  |  |  |  |  |  |  |  |  |  |  |  |
| 2. JX392803 Rodent (*M. glareolus*)/France | 98.2 |  |  |  |  |  |  |  |  |  |  |  |  |  |  |
| 3. LC167302 Human/Netherlands | 98.3 | 99.7 |  |  |  |  |  |  |  |  |  |  |  |  |  |
| 4. EU810406 Human/Germany | 98.3 | 99.7 | 100.0 |  |  |  |  |  |  |  |  |  |  |  |  |
| 5. EU810407 Tick (*I. ricinus*)/Germany | 98.3 | 99.7 | 100.0 | 100.0 |  |  |  |  |  |  |  |  |  |  |  |
| 6. KF849342 Rodent (*M. glareolus*)/Romania | 98.3 | 99.7 | 100.0 | 100.0 | 100.0 |  |  |  |  |  |  |  |  |  |  |
| 7. KF849343 Tick (*I. ricinus*)/Sweden | 98.2 | 99.6 | 99.9 | 99.9 | 99.9 | 99.9 |  |  |  |  |  |  |  |  |  |
| 8. OR225225 Tick (*I. ricinus*)/Spain | 98.3 | 99.7 | 100.0 | 100.0 | 100.0 | 100.0 | 99.9 |  |  |  |  |  |  |  |  |
| 9. KF312363 Tick (*I. ricinus*)/Poland | 98.3 | 99.7 | 100.0 | 100.0 | 100.0 | 100.0 | 99.9 | 100.0 |  |  |  |  |  |  |  |
| 10. KF447527 Tick (*I. ricinus*)/Netherlands | 98.3 | 99.7 | 100.0 | 100.0 | 100.0 | 100.0 | 99.9 | 100.0 | 100.0 |  |  |  |  |  |  |
| 11. EU432375 Canis familiaris/Germany | 98.3 | 99.7 | 100.0 | 100.0 | 100.0 | 100.0 | 99.9 | 100.0 | 100.0 | 100.0 |  |  |  |  |  |
| 12. JQ669946 Tick (*I. ricinus*)/Italy | 98.4 | 99.4 | 99.6 | 99.6 | 99.6 | 99.6 | 99.4 | 99.6 | 99.6 | 99.6 | 99.6 |  |  |  |  |
| 13. KR912350 Rodent (*M. glareolus*)/Slovakia | 98.4 | 99.9 | 100.0 | 100.0 | 100.0 | 100.0 | 99.9 | 100.0 | 100.0 | 100.0 | 100.0 | 99.6 |  |  |  |
| 14. KJ561570 Rodent (*M. glareolus*)/Poland | 98.4 | 99.9 | 100.0 | 100.0 | 100.0 | 100.0 | 99.9 | 100.0 | 100.0 | 100.0 | 100.0 | 100.0 | 100.0 |  |  |
| 15. KJ561571 Human/Poland | 98.4 | 99.9 | 100.0 | 100.0 | 100.0 | 100.0 | 99.9 | 100.0 | 100.0 | 100.0 | 100.0 | 100.0 | 100.0 | 100.0 |  |

⚫, Sequence obtained in the study.
