# Supplementary material for: Detection and phylogenetic classification of Neoehrlichia mikurensis in rodents from the region of Liupan Mountain, China
Source: Front Microbiol. 2024 Jul 4;15:1409593. doi: 10.3389/fmicb.2024.1409593 (PMC11255843; doi:10.3389/fmicb.2024.1409593)
Supplement: Supplementary file 3 [file Table_3.DOCX]

**Supplementary Table 3.** The homologies of PP818814 and sequences of Cluster Ⅲ and Ⅳ in the phylogenetic tree.

|  | 1 | 2 | 3 | 4 | 5 | 6 | 7 | 8 | 9 | 10 | 11 | 12 | 13 | 14 | 15 |
| --- | --- | --- | --- | --- | --- | --- | --- | --- | --- | --- | --- | --- | --- | --- | --- |
| 1. ⚫PP818814 Rodent (*A. peninsulae*)/China: Liupan Mountain |  |  |  |  |  |  |  |  |  |  |  |  |  |  |  |
| 2. AB074461 Tick (*I. ovatus*)/Japan | 95.7 |  |  |  |  |  |  |  |  |  |  |  |  |  |  |
| 3. KU865477 Tick (*I. ricinus*)/Germany | 95.2 | 99.2 |  |  |  |  |  |  |  |  |  |  |  |  |  |
| 4. JQ359067 Rodent (*E. custos*)/China: Yunnan Province | 95.4 | 99.3 | 98.8 |  |  |  |  |  |  |  |  |  |  |  |  |
| 5. JQ359068 Rodent (*A. draco*)/China: Yunnan Province | 95.4 | 99.3 | 98.8 | 100.0 |  |  |  |  |  |  |  |  |  |  |  |
| 6. AB084583 Rodent (*R. norvegicus*)/Japan | 93.8 | 93.1 | 97.4 | 96.7 | 96.7 |  |  |  |  |  |  |  |  |  |  |
| 7. OM648140 Tick (*H. longicornis*)/China: Heilongjiang Province | 93.8 | 96.2 | 97.4 | 96.7 | 96.7 | 99.9 |  |  |  |  |  |  |  |  |  |
| 8. OM648141 Tick (*I. persulcatus*)/China: Heilongjiang Province | 93.8 | 96.2 | 97.4 | 96.7 | 96.7 | 99.8 | 99.9 |  |  |  |  |  |  |  |  |
| 9. JQ359063 Rodent (*N. confucianus*)/China: Fujian Province | 93.8 | 96.7 | 97.3 | 96.7 | 96.7 | 100.0 | 100.0 | 100.0 |  |  |  |  |  |  |  |
| 10. JQ359064 Rodent (*A. agrarius*)/China: Henan Province | 93.8 | 96.7 | 97.3 | 96.7 | 96.7 | 100.0 | 100.0 | 100.0 | 100.0 |  |  |  |  |  |  |
| 11. JQ359065 Rodent (*R. norvegicus*)/China: Henan Province | 93.8 | 96.7 | 97.3 | 96.7 | 96.7 | 100.0 | 100.0 | 100.0 | 100.0 | 100.0 |  |  |  |  |  |
| 12. JQ359066 Rodent (*A. sylvaticus*)/China: Zhejiang Province | 93.8 | 96.7 | 97.3 | 96.7 | 96.7 | 100.0 | 100.0 | 100.0 | 100.0 | 100.0 | 100.0 |  |  |  |  |
| 13. OQ133528 Rodent (*R. tanezumi*)/China: Yunnan Province | 93.8 | 96.6 | 97.2 | 96.5 | 96.5 | 100.0 | 100.0 | 100.0 | 100.0 | 100.0 | 100.0 | 100.0 |  |  |  |
| 14. OQ133533 Rodent (*N. confucianus*)/China: Yunnan Province | 93.8 | 96.6 | 97.2 | 96.5 | 96.5 | 100.0 | 100.0 | 100.0 | 100.0 | 100.0 | 100.0 | 100.0 | 100.0 |  |  |
| 15. OQ875069 Rodent/China: Guangdong Province | 93.9 | 96.5 | 97.1 | 96.4 | 96.4 | 100.0 | 100.0 | 100.0 | 100.0 | 100.0 | 100.0 | 100.0 | 100.0 | 100.0 |  |

⚫, Sequence obtained in the study.
